# Outcome of Endovascular Thrombectomy in Pre-stroke Dependent Patients With Acute Ischemic Stroke: A Systematic Review and Meta-Analysis

**DOI:** 10.3389/fneur.2022.880046

**Published:** 2022-04-28

**Authors:** Antonis Adamou, Androniki Gkana, Georgios Mavrovounis, Eleftherios T. Beltsios, Andreas Kastrup, Panagiotis Papanagiotou

**Affiliations:** ^1^Department of Radiology, Faculty of Medicine, School of Health Sciences, University of Thessaly, Larissa, Greece; ^2^Department of Emergency Medicine, Faculty of Medicine, School of Health Sciences, University of Thessaly, Larissa, Greece; ^3^Department of Neurology, Hospital Bremen-Mitte, Bremen, Germany; ^4^Department of Diagnostic and Interventional Neuroradiology, Hospital Bremen-Mitte/Bremen-Ost, Bremen, Germany; ^5^First Department of Radiology, School of Medicine, National and Kapodistrian University of Athens, Areteion Hospital, Athens, Greece

**Keywords:** acute ischemic stroke, endovascular thrombectomy, pre-stroke disability, systematic review, meta-analysis

## Abstract

**Introduction:**

Endovascular thrombectomy (EVT) is a well-established and effective therapeutic option for patients that meet certain criteria. However, this modality is not well studied in patients with pre-existing disability. The aim of the present study was to investigate the impact of mechanical thrombectomy in patients with acute onset ischemic stroke and pre-stroke dependency (PSD) in regard to their clinical outcome and mortality.

**Materials and Methods:**

The MEDLINE, Scopus, and Cochrane Library databases were comprehensively searched with a cut-off date of December 11th, 2021. We performed meta-analysis to investigate the 90-day clinical outcome, the 90-day mortality, and the rate of symptomatic intracerebral hemorrhage (sICH) between the PSD (modified Rankin Scale score ≥ 3) and non-PSD (modified Rankin Scale score = 0–2) groups who underwent EVT for acute onset ischemic stroke.

**Results:**

Six studies were included in the meta-analysis involving 4,543 cases with no PSD and 591 cases with PSD. The non-PSD group showed a statistically significant better clinical outcome at 90 days compared to the PSD group [RR (95% CI) = 1.44 (1.06, 1.85); *p*_z_ = 0.02]. The non-PSD group demonstrated a statistically significant lower risk of death at 90 days in comparison to the PSD group [RR (95% CI) = 0.45 (0.41, 0.50); *p*_z_ < 0.01]. Lastly, the rate of sICH was comparable between the two groups [RR (95% CI) = 0.89 (0.64, 1.24); *p*_z_ = 0.48].

**Discussion:**

We report a higher rate of unfavorable clinical outcome and a higher mortality rate in patients with PSD undergoing EVT compared to those with no previous disability. However, there was a significant proportion of PSD cases who fared well post-procedurally, indicating that PSD patients should not be routinely excluded from mechanical thrombectomy.

**Systematic Review Registration:**

https://www.crd.york.ac.uk/prospero/display_record.php?ID=CRD42021284181, identifier: CRD42021284181.

## Introduction

The large randomized controlled trials (RCTs) published in 2015 (1–5) have established endovascular thrombectomy (EVT) in stroke patients with large vessel occlusions (6). Their promising findings led to the American Heart Association/American Stroke Association (AHA/ASA) guidelines, recommending that patients ≥ 18 years of age presenting with acute ischemic stroke within 6 h, with an NIHSS score ≥ 6, and a baseline modified Rankin Scale (mRS) score between 0 and 1, may be selected for EVT (7). EVT stands since then as an effective therapeutic approach for patients with large vessel occlusions (8), with the most preferable being the stent retrieval and aspiration techniques, reporting high successful recanalization rates (9). However, the eligibility of patients with prior neurological disability (mRS score ≥ 3) is still unknown since the RCTs that the guidelines were based upon only included patients with mRS scores between 0 and 1 or 0 and 2.

The mRS score evaluates the degree of functional dependence in the performance of daily activities in people with neurological disabilities resulting from stroke or other causes (10), and has become the preferred outcome scale for the majority of relevant clinical trials to date (11). Evidence suggests that individuals with pre-stroke dependency (PSD), assessed by mRS ≥ 3, have a significantly increased risk of institutionalization as well as higher rates of mortality and care costs (12).

EVT is routinely excluded from therapeutic protocols in patients with PSD who present with acute onset ischemic stroke. The aim of the present study was to investigate the impact of mechanical thrombectomy in patients with acute onset ischemic stroke and PSD in relation to clinical outcome and mortality.

## Materials and Methods

### Protocol

The protocol for the current systematic review and meta-analysis was registered on the International Prospective Register of Systematic Reviews (PROSPERO ID: CRD42021284181) and is available in full at: https://www.crd.york.ac.uk/prospero/display_record.php?ID=CRD42021284181.

### Literature Search

Two authors (A.A., A.G.) individually performed the electronic database searches of PubMed (MEDLINE), Scopus (EMBASE), and Cochrane Library databases. The search algorithm included the following terms combined with the Boolean operators “AND” and “OR”: endovascular, mechanical, thrombectomy, treatment, stroke, infarct, pre-stroke, pre-existing, pre-morbid, disability, and dependency. The final literature search was conducted on December 11th, 2021. The references of the retrieved publications were manually screened for relevant articles that were not identified in the original searches. The search algorithms are shown in Supplementary Table 1.

### Data Extraction

Titles, abstracts, and full texts (when necessary) were evaluated for eligibility and irrelevant articles were excluded. Where available, the following variables were extracted: first author; year of publication; male-to-female ratio; mean age; number (n) of cases; n of non-PSD cases prior to intervention; n of PSD cases prior to intervention; n of non-PSD cases post-intervention; n of PSD cases post-intervention; mean/median change of mRS score; n of mRS patients with good clinical outcome at 90 days; n of patients with poor clinical outcome at 90 days; 90-day mortality rate; n of symptomatic intracerebral hemorrhage (sICH) of non-PSD cases; n of sICH of PSD cases; accommodation status prior to intervention; accommodation status post-intervention; occlusion side; M1 segment; M2 segment; large vessel; small vessel; hypertension status; diabetes mellitus status; atrial fibrillation status; coronary disease status; and smoking status, onset to EVT time, onset to reperfusion time. Data extraction was independently performed by two investigators (A.A., A.G.). Discrepancies between the reviewers were resolved by a third investigator (ET.B.).

### Inclusion and Exclusion Criteria

Included studies fulfilled the following predetermined criteria: (a) studies comparing the clinical outcome of mechanical thrombectomy (stent retrieval or aspiration) in patients with PSD (mRS score ≥ 3) treated for acute ischemic stroke vs. patients with no PSD (mRS score 0–2) treated with the same method; and (b) the study types RCTs, non-RCTs, or retrospective cohort studies.

We excluded studies that included patients with mRS scores < 2 and >2 in the non-PSD group (i.e., mRS scores 0–1 and 0–3) and studies with insufficient data regarding the investigated outcome. Finally, we excluded studies with full texts in languages other than English.

### Population, Interventions, Comparators, and Outcomes

All included patients were >18 years of age, undergoing EVT for acute ischemic stroke. Cases were classified into two groups: the “PSD group” included patients with an mRS score = 0–2 and the “non-PSD group” involved patients with an mRS score ≥ 3. All patients underwent thrombectomy, with or without IVT. The primary outcomes were the proportion of cases that achieved a “good clinical outcome” and the 90-d ay mortality rate. The secondary outcome was the incidence of intraprocedural sICH.

### Definitions

“Good clinical outcome” was defined as an mRS score of 0–2 or a return to the pre-stroke mRS score at 90 days. “Poor clinical outcome” was defined as an increase of the mRS score above 2 for the non-PSD group or any increase of the pre-stroke mRS score for the PSD group at 90 days. Post-interventional deaths (mRS = 6) were included in the “poor clinical outcome” group. Mortality was calculated excluding the patients lost at follow-up. Thus, it was defined as the number of post-interventional deaths to the whole number of patients showed up at 90-days. Studies used different criteria for sICH. The criteria suggested by the ECASS III (13–15) and ECASS II (16–18) trials were used by two studies each, while the Heidelberg (19) criteria were used by one study (20). One study did not report any criteria (21). The criteria for sICH are shown in Table 1.

**Table 1:** Criteria used by the included studies for symptomatic intracranial hemorrhage definition.

**Trial**	**Criteria**
ECASS II	Neurological deterioration, regardless of causal factor, or adverse events indicating clinical worsening or causing of increase in NIHSS score of >4 points
ECASS III	Clinical deterioration, predominantly caused by hemorrhage, defined by an increase of >4 points in NIHSS score or led to death
Heidelberg classification scheme	Clinical deterioration defined by an increase of >4 points in NIHSS score at time of diagnosis compared with immediately before worsening or ≥2 points in 1 NIHSS category, or leading to intubation, hemicraniectomy, ventricular rain placement, or other major medical/surgical intervention

*ECASS, European Cooperative Acute Stroke Study; NIHSS, National Institute of Health Stroke Scale*.

### Statistical Analysis

The pooled risk ratios and their 95% confidence intervals (CI) were calculated for the 90-day clinical outcome, the 90-day mortality, and the rate of sICH between the PSD and non-PSD groups. Statistical significance was determined using the Z test, with a value of *p* < 0.05 considered as the significance threshold. A meta-analysis of proportions and their 95% CI was also conducted for the proportion of patients with good clinical outcome at 90 days, in both PSD and non-PSD groups.

Pooled risk ratios or proportions were combined using the appropriate model (random- or fixed-effects) based on the presence of heterogeneity. Cochran's Q and I^2^ indices were calculated for the statistical heterogeneity of the studies to be estimated. The random effects model was applied when *p*_Q_ < 0.10 and/or I^2^ > 50%. Otherwise, the fixed-effects model was used. The minimum number of studies included to perform statistical analysis of the data (meta-analysis) was 3.

Sensitivity analyses were performed to explore whether the statistical analysis (random- or fixed-effects model) or the exclusion of possible outlier studies from each meta-analysis would influence our results. The entirety of the statistical analyses was performed with Review Manager (RevMan) Version 5.3 software [The Nordic Cochrane Centre, The Cochrane Collaboration, Copenhagen, Denmark (http://tech.cochrane.org/revman)], except the proportional meta-analyses, that were performed with the OpenMetaAnalyst for OS X software. Finally, PRISMA guidelines for reporting reviews and meta-analyses were applied (Supplementary Table 2) (22).

### Publication Bias Assessment

Visualization of funnel plots and Begg and Mazumdar (23) and Egger et al.'s (24) tests were used for publication bias assessment, with *p*-values < 0.05 indicating significant publication bias.

## Results

### Selection and Characteristics of the Included Studies

**Figure 1:**
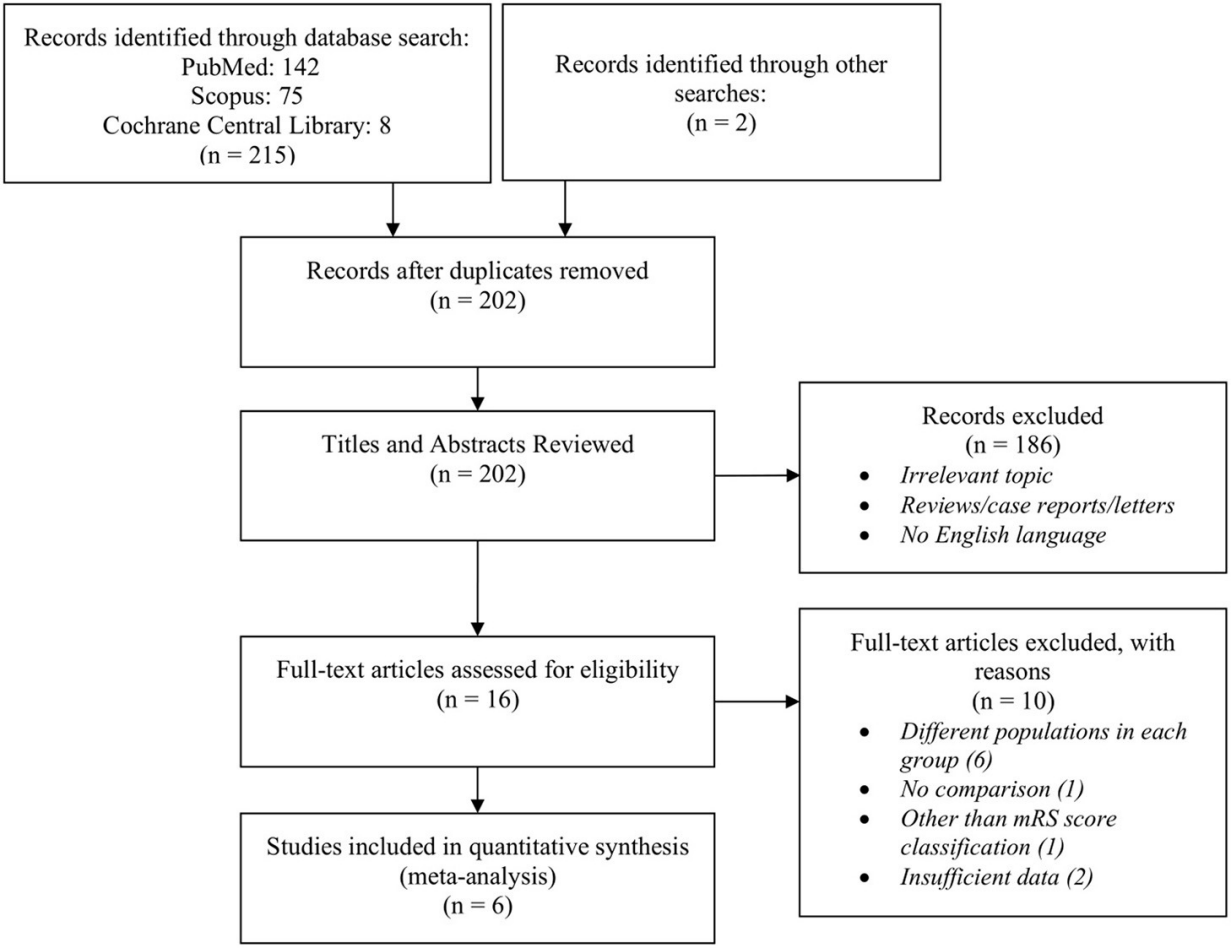
Flowchart of study selection algorithm according to PRISMA guidelines.

The structured search of PubMed, Scopus, and Cochrane Library databases, combined with manual screening of retrieved articles, yielded 217 studies published between 2000 and 2021. Ineligible papers were excluded by reviewing titles and abstracts, resulting in 15 studies suitable for further review. After reading the full-text articles, six studies were excluded due to having included different populations in each group, two studies were excluded for insufficient data regarding the mRS scores of the participants, one study was excluded for not having a comparison group, and one study was excluded for not using the mRS score as the classification score for disability. Finally, six studies published between 2017 and 2021 were included in our meta-analysis (14, 15, 17, 18, 20, 21), involving 4,543 cases with no prior disability and 591 cases with prior functional dependency. Six studies were included for the quantitative analysis of sICH (14, 15, 17, 18, 20, 21), while all but one study (15) were included in the meta-analysis of clinical outcome and mortality. The latter study was excluded due to having a different definition for clinical outcome (mRS score of 4 or higher was defined as unfavorable outcome). The characteristics and the baseline and outcome variables of the included studies are shown in Table 2 and Supplementary Table 3, respectively. The study selection flowchart according to PRISMA guidelines is shown in Figure 1.

**Table 2:** Characteristics of the included studies.

		***n***			**Mean age**			**Outcome *n* (%)**		**Onset to EVT (min)**		**Onset to reperfusion (min)**		
**Study**	**Study type**	**Total**	**Non-PSD**	**PSD**	**Non-PSD**	**PSD**	**M/F ratio**	**Non-PSD**	**PSD**	**Non-PSD**	**PSD**	**Non-PSD**	**PSD**	**Source of included cases**
Leker et al. (15)	Prospective observational study	131	108	23	66.9 ± 14	80.3 ± 10	65/ 66	NA	NA	238 ± 165	209 ± 114	288 ± 176	254 ± 126	A prospectively recruited stroke registry, Hadassah-Hebrew University Medical Center
Goldhoorn et al. (20)	Retrospective analysis	1,441	1284	157	69 (59–78)	80 (71–86)	770/671	491 (38.2)	40 (25.5)	205 (160–265)	220 (165–270)	266 (216–330)	291 (222–338)	MR CLEAN registry, all centers with endovascular treatments in the Netherlands
Oesch et al. (21)	Prospective non-randomized study	1247	1163	84	72 (60–79)	81 (73.75–85)	660/587	516 (44.4)	22 (26.2)	276.5 (199.5–352.25)	272 (209–363)	NA	NA	Bernese stroke center database, University Hospital of Berne, Switzerland
Larsson et al. (14)	Retrospective analysis	591	501	90	74	86	319/272	71 (14.9)	20 (22.7)	NA	NA	NA	NA	Sahlgrenska Stroke Recanalization Registry^a^, Sahlgrenska University Hospital
Nababan et al. (17)	Retrospective cohort study	802	720	82	73 (62–82)	85 (78–89)	422/380	402 (60.5)	31 (37.8)	NA	NA	273 (180–521)	245 (170–363)	Three tertiary hospitals in Perth, Western Australia
Florent et al. (18)	Prospective observational study	922	767	155	67.4 ± 14.8	80.3 ± 12.4	423/499	343 (44.7)	40 (25.8)	234 (188–296)	242 (190–293)	NA	NA	Lille Mechanical Thrombectomy Database, Lille University, France
Total		5,134	4,543	591				1,823 (40.5)	153 (26.7)					

a *Combining data from the Swedish Stroke register, the EVAS-registry, the Regional population registry and the patients' medical records*.

*EVT, Endovascular thrombectomy, PSD, Pre-stroke dependent patients; M/F, Male to female; NA, Not applicable; MR CLEAN, Multicenter Randomized Clinical Trial of Endovascular Treatment for Acute Ischemic Stroke in the Netherlands*.

### Good Clinical Outcome at 90 Days in PSD and Non-PSD Patients

**Figure 2:**
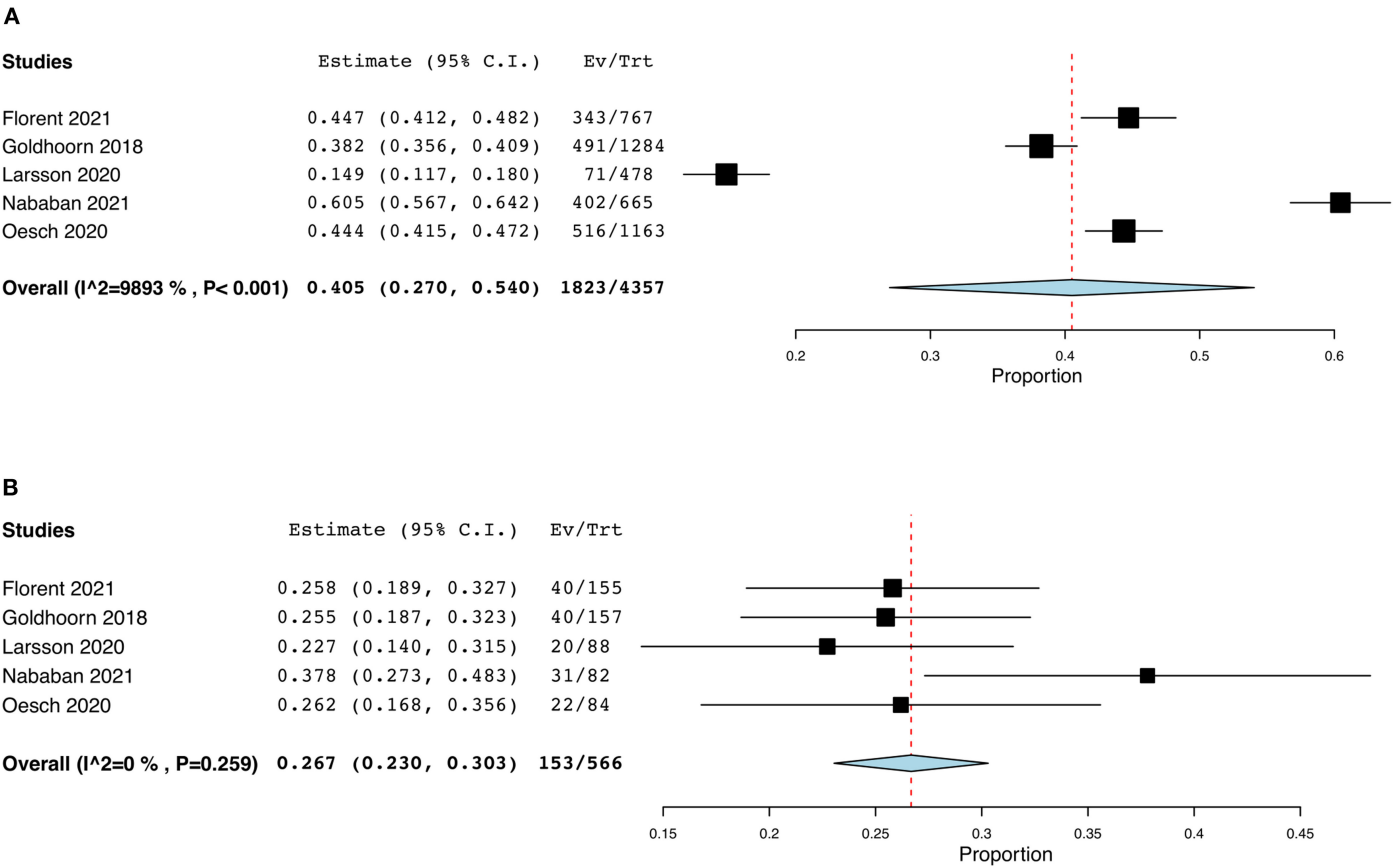
Forest plots: **(A)** proportional meta-analysis of the percentage of patients with good clinical outcome in the non-PSD group, **(B)** proportional meta-analysis of patients with good clinical outcome in the PSD group.

Five studies were included in this meta-analysis. The characteristics of the studies are shown in Table 2. All studies had a retrospective cohort design. Regarding the proportion of patients with good clinical outcome, the random-effects model was applied in the non-PSD group, due to high heterogeneity (I^2^ = 98.9%; *p* = <0.001). The proportion (95% CI) of patients with good clinical outcome in the non-PSD group was 40.5% (27%, 54%) (*p*_*z*_ < 0.001). In the PSD group, the fixed-effects model was applied due to low heterogeneity (I^2^ = 24.4%; *p* = 0.259). The proportion (95% CI) of patients with good clinical outcome in the non-PSD group was 26.7% (23%, 30.3%) (*p*_*z*_ < 0.001). The results of the meta-analysis can be found in Figure 2.

**Figure 3:**
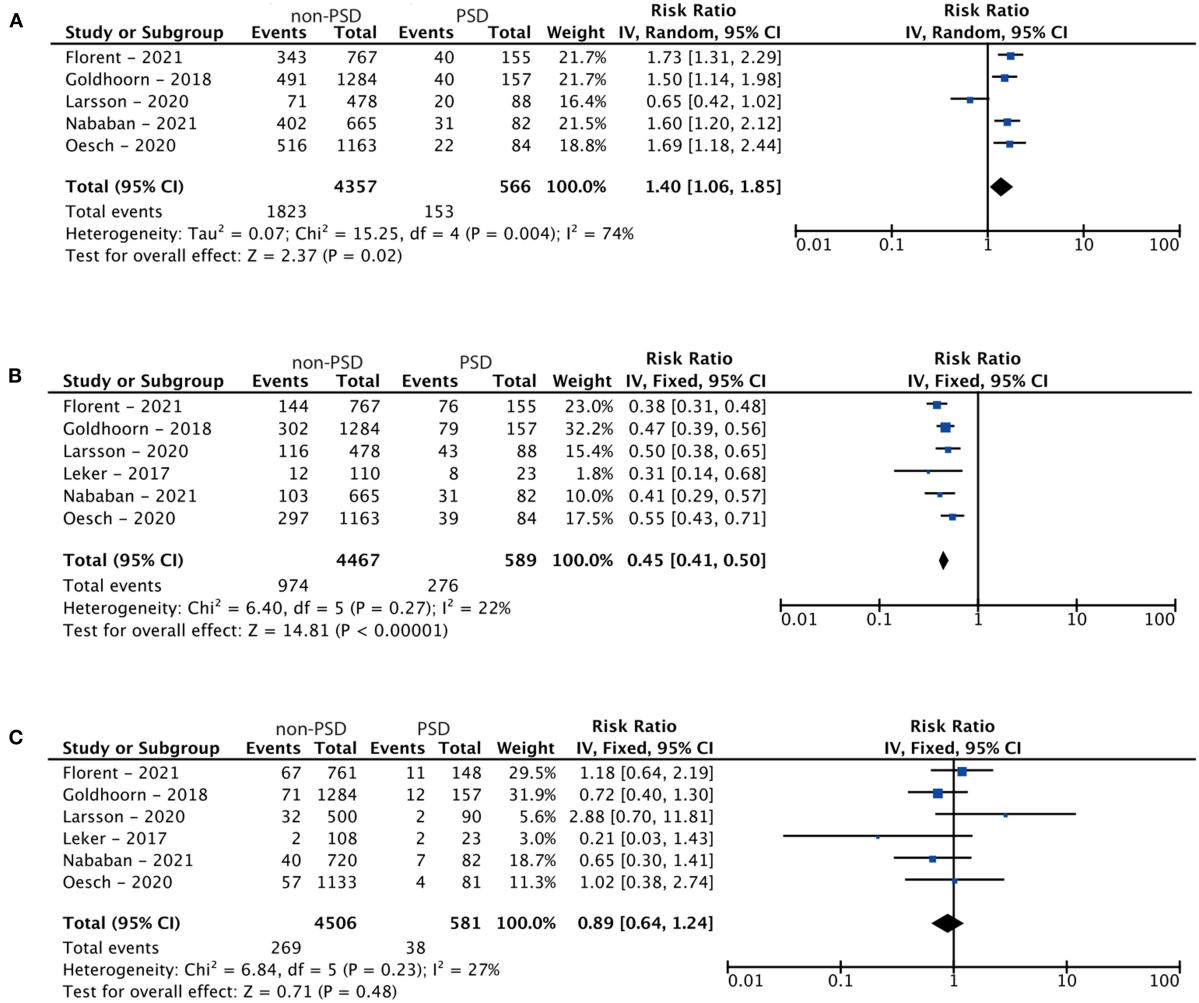
Forest plots: **(A)** meta-analysis of the clinical outcome of endovascular thrombectomy in patients with pre-stroke dependency at 90 days, **(B)** meta-analysis of mortality in patients with pre-stroke dependency undergoing endovascular thrombectomy, and **(C)** meta-analysis of the rate of symptomatic intracranial hemorrhage related to treatment.

Regarding the comparative analysis of the two groups, the random-effects model was applied due to high heterogeneity (I^2^ = 74%; *p* = 0.004). The non-PSD group showed superior results compared to the PSD group in terms of their clinical outcome [RR (95% CI) = 1.40 (1.06, 1.85); *p*_z_ = 0.02]. The results of this meta-analysis are illustrated in Figure 3A.

### Mortality at 90 Days in PSD and Non-PSD Patients

Six studies were included in this meta-analysis. The characteristics of the studies are shown in Table 2. All studies had a retrospective cohort design. Due to low heterogeneity (I^2^ = 22%; *p* = 0.27), the fixed-effects model was applied. With regards to mortality, the non-PSD group demonstrated superior results in comparison to the PSD group [RR (95% CI) = 0.45 (0.41, 0.50); *p*_z_ < 0.01]. The results of this meta-analysis are shown in Figure 3B.

### Symptomatic Intracranial Hemorrhage in PSD and Non-PSD Patients

Six studies were included in this meta-analysis. The characteristics of the studies are shown in Table 2. All studies had a retrospective cohort design. Due to low heterogeneity (I^2^ = 27%; *p* = 0.23), the fixed-effects model was applied. Analysis resulted in no statistical significance [RR (95% CI) = 0.89 (0.64, 1.24); *p*_z_ = 0.48], indicating a comparable rate of sICH between the treatment groups. The results of this meta-analysis are shown in Figure 3C.

### Sensitivity Analysis

Sensitivity analyses based on statistical model selection and the exclusion of possible outlier studies proved the robustness of our results. The results of the sensitivity analysis can be found in Table 3.

**Table 3:** Results of the sensitivity analysis.

**Outcome**	**Sensitivity analysis reason**	**Result**
Good outcome	Changing from random- to fixed-effects To explore if the model choice influenced our results	1.49 (1.30, 1.72) *p* < 0.00001
	Leaving Larsson et al. out as outlier	1.62 (1.40, 1.88) *p* < 0.00001
Mortality	Changing from fixed- to random-effects To explore if the model choice influenced our results	0.45 (0.40, 0.51) *p* < 0.00001
	Leaving Leker et al. out as outlier	0.46 (0.41, 0.51) *p* < 0.00001
sICH	Changing from fixed- to random-effects To explore if the model choice influenced our results	0.89 (0.59, 1.35) *p* = 0.58
	Leaving Leker et al. out as outlier	0.93 (0.66, 1.30) *p* = 0.66
	Leaving Larsson et al. out as outlier	0.83 (0.59, 1.17) *p* = 0.28

### Publication Bias Assessment

Calculation of Begg and Egger *p*-values and funnel plot inspection confirmed no publication bias between the included studies. Funnel plots for both outcomes are shown in Supplementary Figure 1. Begg and Egger *p*-values for both investigated outcomes are illustrated in Supplementary Table 4.

## Discussion

To the best of our knowledge, this is the first meta-analysis to investigate the impact of EVT in patients with ischemic stroke and PSD. Our results suggest a possible difference between patients with and without PSD in terms of their clinical outcome and mortality at 90 days following EVT. The non-PSD group demonstrated a more favorable clinical outcome and a lower risk of death compared to the PSD group. Furthermore, we report a comparable incidence of sICH between the two groups. Compared to those with no previous disability, our findings suggest an unfavorable clinical outcome and a higher mortality rate in PSD patients with disability undergoing EVT; however, there was a significant proportion of PSD cases who fared well post-procedurally.

Our results are in line with the majority of previous reports. According to Florent et al. (18), patients with PSD tend to have an unfavorable outcome compared to patients with no PSD. However, they report a favorable outcome in one out of every four patients, which is a high proportion of patients that would benefit from EVT in terms of their clinical outcome. Therefore, the authors suggest that EVT should not be excluded as a potential therapeutic option merely due to the PSD status alone. The results of our study are comparable since we report 26.7% of patients with PSD return to the baseline mRS score at 90 days. Leker et al. (15) was the only study that did not report positive clinical outcomes in the PSD group. Despite having a study population too small to generate solid conclusions, they conclude that some patients maintained a moderate disability status post-procedurally, thus suggesting the consideration of EVT in all patients with distal internal carotid or proximal middle artery occlusions including patients with moderate disability. Goldhoorn et al. (20) also suggested that PSD patients should not be routinely excluded from EVT since a significant proportion of them will maintain the same pre-stroke mRS score post-procedurally.

There is some disagreement in the literature regarding the comparison groups since some studies define PSD as an mRS score ≥ 2 while others use a ≥ 3 threshold. This inconsistency may arise from the RCT definition of functional independence as an mRS score ≤ 2 even though AHA/ASA guidelines recommend EVT for patients with an mRS score ≤ 1. Future RCTs should pay closer attention to these details so that findings can be more relatable between studies and increase eligibility. Fewer studies have investigated the clinical outcome of EVT in PSD patients with an mRS score ≥ 2 (25–28). Their conclusions were consistent and in agreement with the findings of the present meta-analysis.

Some studies have investigated the impact of EVT or bridging therapy compared to IVT alone in patients with PSD. For example, Kastrup et al. (29) reported that EVT improved clinical outcome at the time of discharge, avoided poor outcome, and caused smaller infarcts compared to IVT in PSD patients. However, more randomized trials and comparative studies that adopt this study design are needed to shed light on whether this group of PSD patients should be recommended for EVT. By doing so, valuable information would be provided for a group of patients that has previously been excluded from large RCTs (1–5).

The number of cases with acute stroke and pre-existing disability is rising with the extension of life expectancy. Caregivers can enhance survival and improve the quality of care in this population by not withholding EVT based upon PSD status alone. Nonetheless, there are some ethical issues to consider when selecting PSD patients for EVT, since extending survival comes at the price of living longer with a severe disability, and possibly bedridden if they achieve an mRS score of 5 post-procedurally. However, the Rehabilitation Act and the Americans with Disabilities Act support the legal prohibition of limited health care services for people with disabilities compared to non-disabled people (30). Therefore, although treatment has the risk of worsening disablement, it should still be considered for patients with disability, particularly in light of the fact that a significant proportion might return to baseline neurological status after EVT. We strongly recommend more extensive studies and RCTs in this area.

The present study is subject to several limitations. Firstly, there are concerns of possible publication bias due to the exclusion of gray literature in our data acquisition strategy. Our publication bias assessment was also indicative of the possible presence of publication bias. In addition, there is increased risk of selection bias since a control group of PSD and non-PSD patients who were not treated with EVT is lacking. Therefore, we cannot draw conclusions about the treatment effect of EVT in both groups. In most registries only data of PSD patients who were “given the benefit of the doubt” were collected and we do not have information about a large group of PSD patients who were not treated with EVT, or were treated with an alternative method (i.e., thrombolysis). Furthermore, the included studies were mostly retrospectively designed, and the number of included subjects was small, thereby limiting the generalizability of the reported results. Therefore, our conclusions should be interpreted cautiously since treatment effects might be overestimated in meta-analyses that include only a small number of participants and published data. Finally, studies written in languages other than English were not included in our study, raising concerns for language bias.

## Conclusions

We report a higher rate of unfavorable clinical outcome and mortality in patients with PSD undergoing EVT compared to those with no previous disability, while the incidence of sICH was found to be comparable between the two groups. Nonetheless, a significant proportion of PSD cases fared well post-procedurally and therefore should not be routinely excluded from mechanical thrombectomy. To support these findings, well-designed and comprehensive RCTs that evaluate EVT should include PSD patients in future studies, and more data is needed on clinical outcomes of EVT vs. IVT in PSD patients.

## Data Availability Statement

The original contributions presented in the study are included in the article/Supplementary Material, further inquiries can be directed to the corresponding author/s.

## Author Contributions

AA and PP: conceptualization. AA, AG, GM, and EB: methodology. AA, AG, and GM: formal analysis. AA: data curation. AA, AK, and PP: writing—original draft preparation and writing—review and editing. AA and GM: visualization. PP: supervision and project administration. All authors contributed to the article and approved the submitted version.

## Conflict of Interest

The authors declare that the research was conducted in the absence of any commercial or financial relationships that could be construed as a potential conflict of interest.

## Publisher's Note

All claims expressed in this article are solely those of the authors and do not necessarily represent those of their affiliated organizations, or those of the publisher, the editors and the reviewers. Any product that may be evaluated in this article, or claim that may be made by its manufacturer, is not guaranteed or endorsed by the publisher.
